# Studies on the concerted interaction of microbes in the gastrointestinal tract of ruminants on lignocellulose and its degradation mechanism

**DOI:** 10.3389/fmicb.2025.1554271

**Published:** 2025-05-09

**Authors:** Runqi Fu, Lin Han, Qian Li, Zhe Li, Yue Dai, Jing Leng

**Affiliations:** ^1^Faculty of Animal Science and Technology, Yunnan Agricultural University, Kunming, China; ^2^Key Laboratory of Animal Nutrition and Feed Science of Yunnan Province, Yunnan Agricultural University, Kunming, China

**Keywords:** lignocellulose, ruminant, gastrointestinal tract, microbes, enzyme

## Abstract

The complex structure of lignocellulose, one of the most abundant renewable resources on earth, makes biodegradation challenging. Ruminant gastrointestinal microbiota achieves efficient lignocellulose degradation through a highly synergistic ecosystem, which provides an important research model for sustainable energy development and high value-added chemical production. This review systematically summarizes the key mechanisms of lignocellulose degradation by ruminant gastrointestinal microorganisms, focusing on the synergistic roles of rumen and hindgut (including cecum, colon, and rectum) microorganisms in cellulose, hemicellulose, and lignin degradation. The study focuses on the functional differentiation and cooperation patterns of bacteria, fungi and protozoa in lignocellulose decomposition, and summarizes the roles of carbohydrate-active enzymes (CAZymes) and their new discoveries under the histological techniques. In addition, this manuscript explores the potential application of gastrointestinal tract (GIT) microbial degradation mechanisms in improving the utilization of straw-based feeds. In the future, by revealing the mechanism of microbe-host synergy and integrating multi-omics technologies, the study of ruminant gastrointestinal microbial ecosystems will provide new solutions to promote the efficient utilization of lignocellulose and alleviate the global energy crisis.

## 1 Introduction

Lignocellulosic waste is one of the most abundant renewable resources on earth and offers significant potential for sustainable production of green energy and high value-added chemicals. It has been estimated that globally about 120 × 10^9^ tons of lignocellulosic biomass is produced annually (Abraham et al., 2020). However, the majority of lignocellulosic resources are currently underutilized, especially in developing countries, and these biomasses are usually disposed of by open burning, leading to severe environmental pollution and posing a threat to human health (Kapoor et al., 2020). Agricultural biomass burning still accounts for about 23% of global biomass burning emissions, despite the fact that lignocellulosic biomass can improve soil quality, increase soil organic matter content, and be used as animal feed (Zhang et al., 2021). Lignocellulosic biomass is highly complex in structure, consisting mainly of cellulose (40–60%), hemicellulose (20–40%) and the aromatic polymer lignin (10–25%) (Kumar et al., 2021; Tye et al., 2016). However, its inherent physical and chemical properties (e.g., crystallinity of cellulose, degree of polymerization, and protective barrier of lignin) contribute dramatically to the difficulty of its biodegradation, thus limiting its potential for application in biofuel and chemical production (Bhujbal et al., 2021; Xu et al., 2021). To overcome these challenges, different pretreatment methods have been proposed, including physical (e.g., grinding and irradiation), chemical (e.g., treatment with acids, bases, oxidizers, and organic solvents), physicochemical (e.g., extrusion, hydrothermal, and vapor explosion), and biological (e.g., microbial and enzymatic treatments) approaches (Dutta et al., 2021; Yu et al., 2020). Nevertheless, each of these techniques has some limitations. For example, chemical pretreatment is costly and prone to produce by-products that inhibit methanogenesis; some of the methods are energy intensive, making it difficult to strike a balance between economic and environmental sustainability (Kumar et al., 2021).

The ruminant has succeeded in achieving efficient degradation of lignocellulose through a complex and highly synergistic microbiota in the gastrointestinal tract (GIT). This ability is derived from the action of microbial symbionts, as the ruminant’s own genome lacks genes encoding hydrolytic enzymes required for the degradation of polysaccharides in plant cell walls (Mizrahi and Jami, 2021). In recent years, remarkable progress has been made in studies targeting the rumen of ruminants. It has been shown that rumen microbiota not only efficiently degrade lignocellulose, but also considerably improve the methane production of biomass through rumen fluid pretreatment. For example, rumen fluid pretreatment of rice straw increased methane production by 82.6% (Zhang et al., 2018). In addition, rumen microbiota has been found to degrade 41.23–82.23% of volatile solids (VS) and achieve methane yields ranging from 287 to 310.25 mL per gram of VS under conditions that do not require physical or chemical pretreatment (Candia-García et al., 2018; Thongbunrod and Chaiprasert, 2021). The efficiency of lignocellulosic biomass degradation by the bovine rumen is approximately three times higher compared to conventional anaerobic digesters (Bhujbal et al., 2021). Regardless of the dominant role of the rumen in lignocellulosic degradation, recent studies have gradually recognized the important contribution of the hindgut microbiota. While rumen microorganisms mainly decompose easily fermentable feed components, hindgut microorganisms specialize in the processing of undigested crystalline cellulose in the foregut, especially in the cecum and colon, which are enriched with microorganisms that degrade crystalline cellulose such as *Clostridium* (Xie et al., 2021; Jiang et al., 2022; Yan et al., 2022; Cao et al., 2023; Wu et al., 2024). Studies have shown that the hindgut (includes the cecum, colon and rectum) is the second largest fermentation organ in ruminants after the rumen, where about 30% of fiber, xylose and hemicellulose are fermented and degraded (Zhu et al., 2021). In addition, the microbiota in the colon is an efficient utilizer of undigested food residues, providing about 50% of the host’s energy requirements (Bugda Gwilt et al., 2020). Therefore, in-depth studies on the role of ruminant gastrointestinal microbes in lignocellulose degradation not only provide important insights into their unique digestive ecological mechanisms, but also lay the groundwork for the development of industrially relevant enzymes (e.g., cellulases, hemicellulases, and ligninases) and biofuel (e.g., biohydrogen and biogas) production technologies. These studies were significant in promoting the sustainable utilization of lignocellulosic resources and alleviating the current global energy and environmental crises. With the advancement in microbiomics technology, researchers are able to explore unculturable gastrointestinal microorganisms in depth and apply them to the field of biotechnology.

Although the role of rumen microbes in lignocellulose degradation in ruminants has been previously investigated (Fliegerova et al., 2015; Mizrahi and Jami, 2018; Moraïs and Mizrahi, 2019; Thapa et al., 2020), the present study advances knowledge in this area in several ways. Firstly, in terms of microbial community research, the present study provides a more in-depth and comprehensive analysis, not only detailing the role of rumen microbes, but also highlighting the importance of hindgut microbes in lignocellulosic degradation, which may not have been sufficiently focused on in some previous reviews. Secondly, this study followed the latest development of multi-omics technologies and elucidated how these technologies can be applied to deeply analyse the structure and function of microbial communities, providing new perspectives and methods for future research, which may not have been adequately reflected in previous reviews. In addition, this study has also explored the synergistic mechanism of microbial enzymes and their potentials in practical applications, providing theoretical basis for the development of highly efficient degradative enzymes and biomimetic anaerobic digestive systems, which are important additions and extensions to the previous reviews.

## 2 Lignocellulose composition and structure

**Figure 1:**
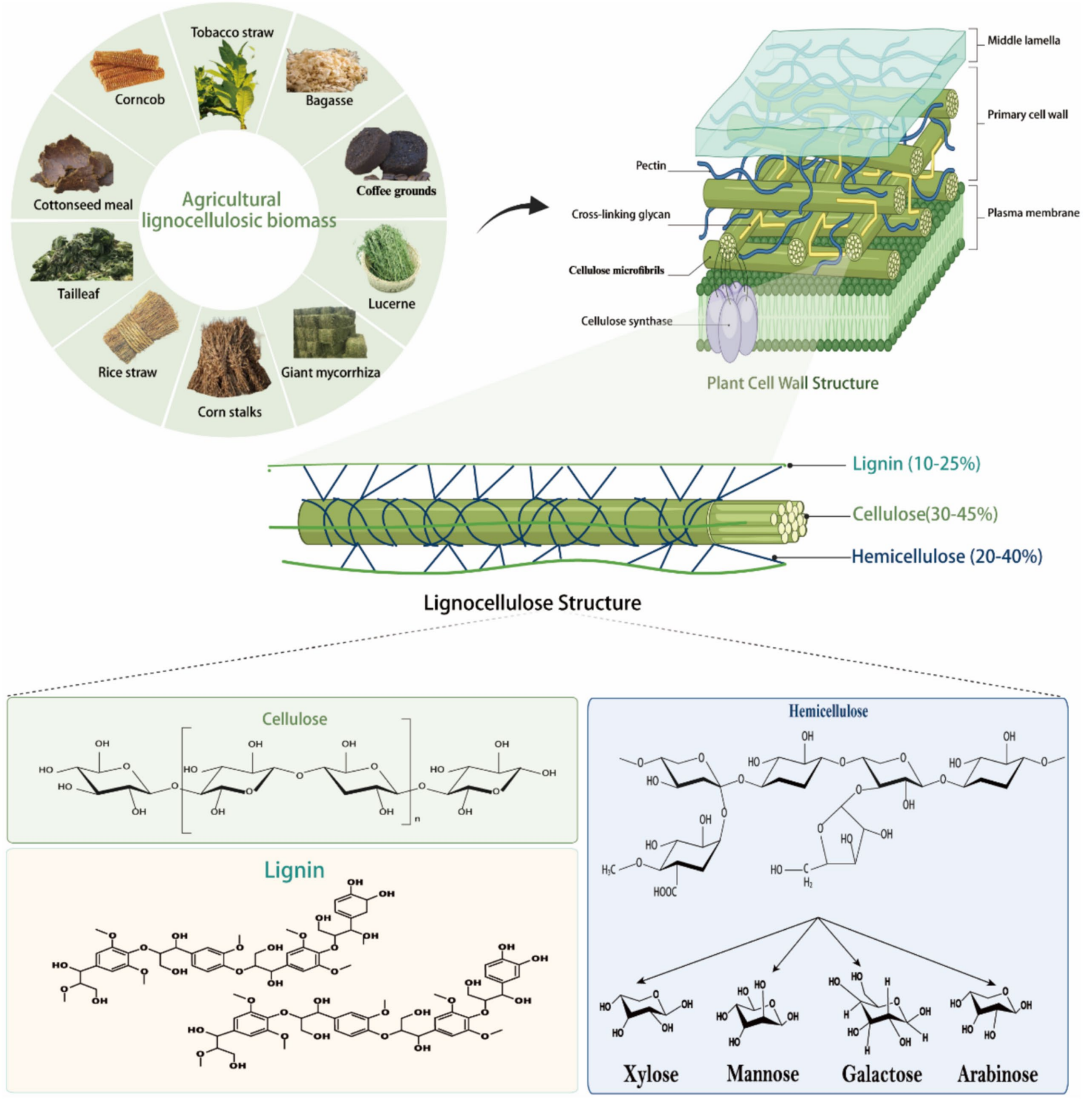
Composition and structure of lignocellulose. Lignocellulose, a highly recalcitrant composite, consists of three primary polymers: cellulose, hemicellulose, and lignin. Cellulose is a homopolymer of D-glucose linked by β-(1 → 4)-glycosidic bonds, with repeating cellobiose units stabilized by hydrogen bonding into long, rigid chains (Estela and Luis, 2013). Hemicellulose is a heteropolymer of monosaccharides (e.g., xylan, mannose, galactose, arabinose) that forms chemical bonds with lignin and hydrogen bonds with cellulose (Zhou et al., 2017; Yang et al., 2015). Lignin is composed of coniferyl alcohol, *ρ*-coumaryl alcohol, and kaempferol, connected via C-C and ether bonds, contributing to the structural rigidity of the matrix (Schutyser et al., 2018). Together, these polymers form the robust architecture of lignocellulose.

Lignocellulose is a renewable and sustainable biomass resource and is a major component of plant cell walls (Abraham et al., 2020). It consists of major cellulose, hemicellulose, lignin, and small amounts of pectin, proteins, minerals, chlorophyll extracts, waxes, and non-structural sugars, making it a complex, highly resistant, and difficult to degrade biomass (Figure 1; Xing et al., 2020). The content of cellulose, hemicellulose, and lignin varies depending on the plant species, developmental stage, and tissue, with the cell wall containing an average of 40–60% cellulose, 20–40% hemicellulose, and about 20% lignin (Tye et al., 2016). Cellulose and hemicellulose are polysaccharides composed of carbohydrate monomers, whereas lignin consists of nonrepetitive hydrophobic aromatic units of different structures. Unlike digestible cell wall polysaccharides, lignin cannot be digested under anaerobic conditions in the rumen and thus acts as a physical barrier to gastrointestinal microorganisms and their enzymes.

### 2.1 Cellulose structure

Cellulose forms chains of 500–15,000 D-glucans interconnected by β-1,4-glycosidic bonds (Figure 1; Gharechahi et al., 2023). During cellulose biosynthesis, every 50–100 cellulose chains combine to form one primitive fibril with a diameter of 4 nm, and about 20 primitive fibrils aggregate to form microfibrils with a crystalline structure that is visible under electron microscopy (Gharechahi et al., 2023). The cellulosic chains bound together by intra- and intermolecular hydrogen bonds, forming microfibrils 3 of 2–20 nm in diameter and 100–40,000 nm in length (Sethi et al., 2013). The cellulose molecule has a “reducing end” with a hemi acetal exhibiting the reducing aldehyde and a “non-reducing end” where the C1 carbon of the hemiacetal cycle is involved in the β-(1,4)-glyco sylic bond (Ghose, 1987). Cellulose is divided into crystalline and amorphous regions, with the crystalline regions characterized by tight intermolecular connections via hydrogen bonds and van der Waals forces, rendering them resistant to degradation (Estela and Luis, 2013). In contrast, the amorphous regions exhibit weaker intermolecular bonds, making them more susceptible to enzymatic breakdown. It has been found that cellulose polymers are degraded by cellulases, which cleave the β-1,4 bond in the cellulose chain. Cellulases can be classified into three groups based on their mode of action: endo-cellulases randomly cleave the cellulose chain in the internal amorphous region, exo-cellulases or cellulose biohydrolases (reducing and non-reducing ends) cleave the cellulose chain from the ends and release the disaccharide fiber biosaccharides, and fiber biotinases or β-glucosidases cleave the β-1,4 linkages of the two consecutive D-glucose units in fiber biosaccharides (Thapa et al., 2020). Based on sequence similarity in the Carbohydrate Active Enzyme (CAZy) database,^1^ cellulose-degrading enzymes have been classified into different glycoside hydrolase (GH) families, including GH1, GH3, GH5, GH6, GH8, GH9, GH12, GH45, GH48, GH51, and GH74 (Drula et al., 2022). Since non-cellulolytic microorganisms are unable to digest other polysaccharides in the presence of cellulose, the ability to degrade cellulose is critical for the digestion of plant cell wall polysaccharides in the gastrointestinal tract.

### 2.2 Hemicellulose structure

Hemicellulose, the second most abundant component of lignocellulose, comprises complex, branched polysaccharides with one or two types of sugar monomers (Figure 1; Yang et al., 2015). Unlike linear cellulose, hemicellulose forms hydrogen bonds with cellulose, enhancing plant cell wall integrity (Prajapati et al., 2018). Hemicellulose is categorized into xylans, mannans, galactans, arabinoxylans, ferulic acid esters, and xyloglucans based on its primary sugar residues (Figure 1; Scheller and Ulvskov, 2010). Xylans, the most prevalent hemicellulosic polysaccharides, feature a β-1,4-linked xylose backbone with side chains of arabinose, galactose, glucose, glucuronic acid, 4-O-methyl-D-glucuronic acid, and additional xylose residues. They are further classified into glucuronosylated xylans, arabinoxylans, and glucuronosylated arabinoxylans based on side chain structure and acetylation levels (Zhou et al., 2017). Mannans, another major hemicellulose type, have a β-1,4-linked mannose backbone with α-1,6-linked galactose in galactomannans or unbranched in glucomannans. Acetylation of mannans enhances their solubility and enzymatic degradation. Xylose glucans consist of a β-1,4-linked glucose chain with α-1,6-linked xylose side chains, which may be further acetylated or substituted with galactose, fucose, or arabinose (Sethi et al., 2013). Typically, β-1,4-linked glucose residues are spaced with individual β-1,3-linked glucose units to form fibrilotriosyl and fibrilotetraosyl chimeras (Burton and Fincher, 2009). Arabinogalactans, present in lower abundance, are classified into types I and II: Type I has a β-1,4-linked galactose backbone with α-1,5-linked arabinogalactan side chains, while Type II features a β-1,3-linked galactose backbone with possible β-1,6-linked branches (Houfani et al., 2020). Moreover, galactose side chains may contain α-1,6-linked 4-O-methyl-D-glucuronic acid or L-arabinofuranose units. Pectin, an important polysaccharide of plant cell walls, has about 65% of its structure as a linear polymer of α-1,4-linked galacturonic acid, with backbone residues that may be further methylated or acetylated, depending on the plant species (Mohnen, 2008). The arabinose consists of 1,2 or 1,3-linked arabinose residues and/or α-1,5-linked arabino-oligosaccharide branched chains. Galactosaccharides are homopolymers of β-1,4-linked D-galactose residues, possibly further branched through β-1,6-linked galactose or arabinose. The complex structure of pectin requires the synergistic action of hydrolytic and lytic enzymes for effective degradation. Enzymatic degradation of hemicellulose is essential to improve the digestibility and nutritional value of ruminant feeds. An in-depth understanding of the structural diversity of hemicellulose and its microbial degradation pathways can help to optimize the conversion efficiency of plant biomass to nutrients available to ruminants.

### 2.3 Lignin structure

Lignin, a complex, water-insoluble, non-crystalline polymer, consists of coumaryl alcohol, coniferyl alcohol, and sinapyl alcohol, linked irregularly by C-C and C-O bonds (Figure 1; Schutyser et al., 2018). The relative content of these units varies depending on the plant species and extraction method. The assembled lignin units form racemic macromolecules through free radical polymerization, mainly consisting of guaiacol, butyl and p-hydroxyphenyl substituents. The lignin linkage network consists of C-C carbon–carbon bonds (β-β, β-5, 5–5, and β-1) and C-O-C ether bonds (α-O-4, β-O-4, and 4-O-5), which are randomly arranged to reinforce the overall structure of the aromatic unit (Brandt et al., 2015). The lignin structure is primarily stabilized by β-O-4 aryl ether bonds, though these are less stable compared to the robust C-C bonds, particularly the biphenyl 5–5 bonds (Fagerstedt et al., 2015). Furthermore, lignin is closely associated with hemicellulose and/or cellulose by covalent bonds (Shirkavand et al., 2016), ester and hydrogen bonds (Sun et al., 2018). All major structural components in cellulose - hemicellulose - lignin are responsible for the recalcification properties of lignocellulose. Lignin is the main difficult-to-degrade component and serves as a plant defense against its breakdown by hydrolytic enzymes produced by plant pathogens (Bomble et al., 2017). Cellulose is insoluble due to its complex, hard, extremely crystalline and insoluble properties (Nitsos et al., 2019). Hemicellulose restricts access to cellulose and is thus insoluble (Donev et al., 2018). In addition, lignin contains covalent bonds (Lig nin-carbohydrate complexes) between lignin and carbohydrates, mainly hemicellulose, which is a key factor in the recalcification of plant biomass (Tarasov et al., 2018). For example, some hemicelluloses containing arabinoxylans (e.g., graminoids) attach to ferric ferulate, which in turn binds to lignin, resulting in additional recalcification (Yang et al., 2011).

## 3 Physiologic structural advantages of the GIT in ruminants

The GIT of ruminants is composed of 10 compartments: rumen, reticulum, flap stomach, rumen, duodenum, jejunum, ileum, cecum, colon, and rectum. Each compartment is specific in spatial distribution and is regulated by factors such as substrate availability, digestive fluid retention time and pH (Russell and Rychlik, 2001), which markedly affects the composition and function of the microbial community, and consequently physiological processes such as digestion, immunity, metabolism and endocrinology (Martinez-Guryn et al., 2019). The rumen, as the largest compartment, accounts for about 80% of the stomach volume, and its vast capacity and complex microbial ecosystem provide favorable conditions for efficient cellulose catabolism (Olijhoek et al., 2022). In adult cattle, the small intestine consists of the duodenum, jejunum and ileum and is approximately 7–8 m in length; the hindgut consists of the cecum, colon and rectum and is approximately 5–5.6 m in length (van Gastelen et al., 2021). The digestive process begins in the rumen and passes sequentially through the reticulum, the flap stomach and the rumen. In the rumen, microbial fermentation and mechanical agitation function synergistically to breakdown crude fiber into absorbable nutrients. Since ruminants lack the genes for hydrolytic enzymes required to break down plant cell wall polysaccharides (Mizrahi and Jami, 2021), they have evolved specialized rumen and hindgut, which form a symbiotic microbial fermentation system that degrades lignocellulose, provides substrate and energy, promotes the growth of beneficial bacteria and efficiently converts proteins to meet nutritional requirements (Liu et al., 2023). The small intestine increases the absorptive surface area through villi to enhance the efficiency of nutrient absorption (Liu et al., 2023). However, cattle are less than 50% efficient at digesting low-quality forages (McCartney et al., 2006), and concentrate supplementation is often required during feeding to meet growth requirements. Nutritionally, the degradability of cellulose and the frequently underestimated hemicellulose is intermediate between that of the easily degradable pectin and the indigestible lignin, and directly affects the digestibility and nutritional value of the diet. Although cellulose degradation is dependent on enzymatic processes, it is difficult to fully understand its biodegradation without incorporating mechanisms at the cellular and subcellular levels.

### 3.1 Microbial composition of the GIT of ruminants

The GIT of ruminants hosts an exceptionally diverse microbiota, comprising over 1,800 genera and 40,000 bacterial species—totaling billions of bacteria and accounting for 67% of all bacteria in the digestive system, with bacterial cells outnumbering host cells by 10- to 100-fold (O'Hara et al., 2020). These microorganisms are distributed across the GIT, with the highest concentrations found in the rumen, cecum, and colon, where they collaborate to degrade lignocellulose and produce essential nutrients such as volatile fatty acids, proteins, and vitamins to sustain the host. Microbial diversity varies among GIT compartments. Mao et al. (2015) observed that the foregut (rumen, reticulum, omasum) harbors the highest microbial diversity, followed by the hindgut (cecum, colon, rectum), while the small intestine (duodenum, jejunum, ileum) contains comparatively fewer microbes. As digestion progresses from the rumen to the hindgut, environmental acidity and oxygen levels decline, driving a marked increase in microbial abundance (Liu et al., 2023). Bacteria dominate these communities, with concentrations ranging from 10^9^–10^10^ bacteria/mL in the small intestine to 10^12^–10^14^ bacteria/mL in the hindgut. The predominant phyla include Firmicutes, Bacteroidetes, Actinobacteria, and Verrucomicrobia (Liu et al., 2023). Notably, Firmicutes and Bacteroidetes are abundant in the rumen, while the colon is dominated by Firmicutes, and the small intestine primarily contains Firmicutes and Proteobacteria (Mao et al., 2015). These compartmentalized microbial communities exhibit distinct functional roles. Multi-omics studies (Xie et al., 2021; Jiang et al., 2022; Yan et al., 2022; Cao et al., 2023; Wu et al., 2024) reveal that rumen microbes preferentially ferment easily degradable feed components, whereas hindgut microbes specialize in breaking down crystalline cellulose indigestible in the foregut. This function is particularly evident in the cecum and colon, which are enriched with Clostridium species capable of degrading crystalline cellulose. In spite of the synergistic roles of these microbial communities in feed degradation and nutrient supply, most studies to date have focused on microbial taxonomy and function. A deeper understanding of the intrinsic mechanisms of microbial-host interactions remains a critical research gap (Table 1).

**Table 1:** Distribution and number of microorganisms in the GIT of ruminants.

Position	Dominant bacterium	Abundances	Functionality	Source
Rumen	Firmicutes, Bacteroidetes, Proteobacteria	10^10^–10^11^cells/mL	Provides 70% of energy needs through fermentation	Zhu et al. (2022), Kim et al. (2022), and Wang et al. (2022)
Hindgut	Firmicutes, Bacteroidetes, Actinobacteria, and Verrucomicrobia	10^12^–10^14^cells/mL	Fermented fibers meet 30% of the host’s energy needs	Abeyta et al. (2023) and Bach et al. (2017)

#### 3.1.1 Rumen microbial composition of ruminants

The rumen of ruminants hosts a diverse microbial ecosystem primarily consisting of bacteria, fungi, protozoa, and archaea. Bacteria dominate, comprising approximately 95% of the microbial community, whereas archaea account for 2–5%, and eukaryotic protozoa and fungi represent 0.1–1% (Kim et al., 2022; Mizrahi and Jami, 2021; Cao et al., 2023; Wu et al., 2024). These bacterial populations are essential for lignocellulose degradation (Wallace et al., 2019; Zhu et al., 2022), with Firmicutes and Bacteroidetes forming the core community, making up over 80% of the total microbes (Henderson et al., 2015). Rumen fungi, predominantly anaerobic *Novozygomycota*, possess extensive carbohydrate-active enzyme gene pools and complex mycelial structures, enabling effective lignocellulose breakdown (Gruninger et al., 2014; Haitjema et al., 2014; Mao et al., 2015; Xie et al., 2021; Mizrahi and Jami, 2021; Wu et al., 2024). Although archaea are less numerous and diverse, they play a vital role in regulating rumen hydrogen partial pressure and facilitating methanogenesis, thereby maintaining healthy fermentation processes (Mizrahi and Jami, 2018). Recent research indicates that while different ruminant species, such as beef cattle, dairy cows, and goats, share a core microbial group, their rumen microbiota also exhibit species- and diet-specific variations (Wang et al., 2013; Cao et al., 2023; Guo et al., 2024). For instance, high-fiber diets enhance the prevalence of Bacteroidetes, which are enriched in glycoside hydrolases (GHs) and polysaccharide-degrading enzymes that efficiently dismantle complex plant cell wall polysaccharides (Naas et al., 2018). Amplicon sequencing analyses have revealed that the rumen microbial community comprises eight phyla, representing 99% of 16S rRNA sequences (Henderson et al., 2015). Despite variations among ruminants, Firmicutes and Bacteroidetes consistently dominate the core bacterial community due to their robust production of CAZymes (Henderson et al., 2015; La Rosa et al., 2022; McKee et al., 2021; Wu et al., 2024). Additionally, less abundant microbes such as Fibrobacteria, Spirochaetes, Verrucomicrobia, Proteobacteria, Tenericutes, Synergistetes, and Planctomycetes fulfill unique functional roles in cellulose and hemicellulose degradation under different dietary conditions (Gharechahi et al., 2021; Cao et al., 2023; Wu et al., 2024).

#### 3.1.2 Intestinal microbial composition of ruminants

**Figure 2:**
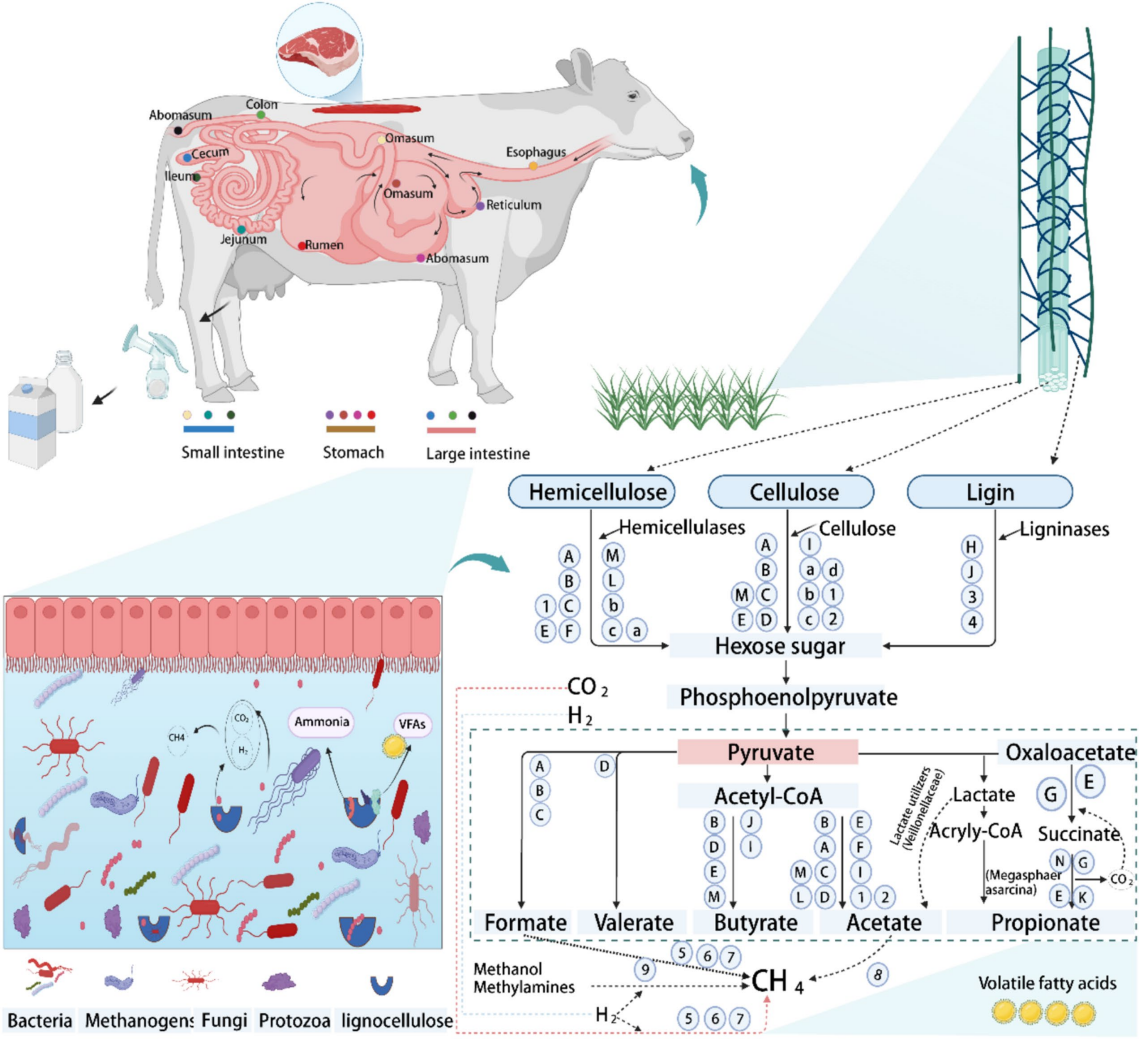
The schematic diagram of the digestive strategy of the gastrointestinal tract (GIT)in ruminants, using cattle as an example. Degradation and metabolic pathways of lignocellulose by peristalsis and microorganisms in the GIT, with special reference to lignocellulosic feeds. Bacterial group: A, *Ruminococcus*; B, *Butyrivibrio*; C, *Fibrobacter*; D, *Clostridium*; E, *Prevotella*; F, *Treponema*; G, *Succiniclasticum*; H, *Pseudomonas*; I, *Eubacterium*; J, *Olsenella*; K, *Rikenellaceae_RC9_gut_group*; L, *Caproiciproducens*; M, *Bacteroides*; N, *Selenomonas*. Fungi group: 1, *Neocallimastix*; 2, *Pecoramyces*; 3, *Ascomycota*; 4, *Basidiomycota*. Protozoa group: a, *Epidinium*; b, *Polyplastron*; c, *Eudiplodinium*; d, *Dasytricha*. Archaeal group: 5, *Methanobrevibacter*; 6, *Methanoculleus*; 7, *Methanosphaera*; 8, *Methanothrix*; 9, *Methanomassiliicoccus*. Conceptualization and modification derived from Liang et al. (2020, 2021), Wardman et al. (2022), Newbold et al. (2015), Abraham et al. (2020), Asanuma and Hino (2001), and Liu et al. (2023).

The gut microbiota of ruminants, comprising the duodenum, jejunum, ileum, cecum, colon, and rectum, mirrors that of monogastric animals in structure but harbors a complex microbiota critical for nutrient metabolism. This ecosystem, notably influenced by the gut’s architecture and the microbial residence time facilitated by the GIT’s large cross-sectional area, plays a crucial role in the thermogenesis and metabolic activity that regulates host energy metabolism (Fan et al., 2019; Liu et al., 2023). While the small intestine primarily undertakes chemical digestion, the large intestine—comprising 14% of the rumen’s volume—serves as the main site for microbial digestion, allowing prolonged microbial fermentation due to its slowed emptying rate. The retention time of chyme is shorter in the small intestine under peristaltic action, whereas in the large intestine it provides a longer time for microbial fermentation due to slower emptying. Although most of the microbial fermentation and digestive activity occurs in the rumen and small intestine, the indigestible cellulose is dependent on the microbiota in the large intestine for catabolism (Liu et al., 2023). Recent studies have shown that the hindgut (cecum, colon and rectum) of ruminants plays a key role in the fermentation and breakdown of fiber, xylose and hemicellulose. Compared to the rumen, the microbiota of the hindgut has higher starch and ADF (acid detergent fiber) digestibility (Kong et al., 2005) and a higher microbial abundance (in terms of the proportion of dry matter of the digestive tract contents). The hindgut microbiota is the second largest fermentation organ in ruminants after the rumen, where about 30% of fiber, xylose and hemicellulose are fermented and degraded, suggesting that the hindgut is crucial in the degradation of lignocellulose. Furthermore, the microbial community in the colon contents efficiently utilizes undigested food residues to produce approximately half of the energy required by the host (Bugda Gwilt et al., 2020). It has been shown that unique microbes in the colon, such as Bifidobacterium, Prevotella, and Ruminococcaceae, play a central role in starch degradation (Jin et al., 2018; Jin et al., 2024). Despite similarities among microbial communities across different gut segments, the cecum and colon show the highest similarity, indicating a functional linkage (Turnbaugh et al., 2006). These segments feature a high microbial diversity, predominantly consisting of Firmicutes, Proteobacteria, and Bacteroidetes, which are crucial for gut health and nutrient absorption (Liu et al., 2023; Jin et al., 2024). Additionally, these microbes produce a vast array of metabolic enzymes exceeding those generated by the host’s own tissues, such as the liver. Notably, certain bacteria are adept at degrading hemicellulose, an otherwise indigestible component in the rumen, and transforming it into nutrients absorbed by the intestinal mucosa for systemic distribution (Zhu et al., 2021; Jin et al., 2018; Jin et al., 2024). While research on the rumen microbiota has advanced, studies on the hindgut microbiota remain limited but are crucial for deepening our understanding of nutrient metabolism mechanisms in ruminants. Further exploration of the hindgut microbiota’s composition and its role in lignocellulose degradation is imperative (Figure 2).

## 4 Degradation patterns of lignocellulose by GIT microorganisms in ruminants

Ruminants rely heavily on fibrous diets, especially in regions with limited or poor-quality forage, to fulfill their energy needs (Liang et al., 2020; Hagen et al., 2021). The GIT of ruminants hosts a complex ecosystem of diverse microorganisms, including bacteria, archaea, anaerobic fungi, and protozoa, which have evolved to efficiently degrade lignocellulose through synergistic interactions and functional differentiation (Mao et al., 2015; Cao et al., 2023; Jin et al., 2024; Wu et al., 2024; Zhu et al., 2021). Approximately 80% of lignocellulose degradation is attributed to bacteria and anaerobic fungi, while protozoa account for the remaining 20% (Basak et al., 2022). Microbial fermentation in the rumen and hindgut, crucial for plant fiber breakdown, generates volatile fatty acids (VFAs), providing up to 70% of a ruminant’s dietary energy (Bergman, 1990). Degradation of plant cell walls relies on the synergistic action of microbial communities, which are functionally differentiated through the formation of unique ecological niches. Despite the uneven distribution of microorganisms in the gut, specific microbial communities in the rumen and hindgut ensure efficient lignocellulose degradation through cooperation (Hou et al., 2024; Jin et al., 2024; Wu et al., 2024). Although archaea do not have the ability to degrade lignocellulose per se, but indirectly influence the fiber degradation process by producing methane from fermentation products (e.g., H₂, acetate, and formate) and have an important impact on energy metabolism and environmental methane emissions in ruminants (Mizrahi and Jami, 2021). While ciliate protozoa possess an array of GHs genes that potentially degrade lignocellulose, their exact roles remain undefined (Williams et al., 2020). Conversely, anaerobic fungi show high potential in degrading tough lignocellulose, albeit at a lower diversity and abundance than bacteria. Recent metaproteomic and metatranscriptomic studies have shown that anaerobic fungi are not only directly involved in cellulose degradation, but also enhance bacterial degradation of hemicellulose through the secretion of exonucleases and endonucleases of the GH5, GH6, GH8, and GH48 families (Hagen et al., 2021; Gruninger et al., 2014). Cellulose is one of the most difficult polysaccharides to degrade in the plant cell wall, and its breakdown requires the synergistic action of a variety of glycoside hydrolases encoded by microbial genomes, reflecting the complex microbial dynamics of the rumen ecosystem (Andersen et al., 2023). Although predicting microbial community dynamics and interactions is complex, ongoing *in vitro* research in co-cultures and microbial consortia is elucidating these mechanisms. Enhanced understanding of GIT fiber-degrading microbes and their community interactions is pivotal for improving roughage utilization in ruminants, enhancing feed efficiency, and potentially reducing methane emissions to mitigate environmental impacts.

### 4.1 Degradation of lignocellulose by bacteria in the GIT of ruminants

The degradation of lignocellulose critically depends on the effective colonization of roughage by gastrointestinal bacteria, with microorganisms that form tight biofilms playing a central role in this process. The dominant bacterial communities in the GIT are composed of Bacteroidetes and Firmicutes, which together account for over 80% of the microbial population in the rumen, highlighting their significant role in rumen functionality (Jin et al., 2024). Biofilm formation, a common characteristic of most lignocellulose-degrading bacteria, not only facilitates bacterial adhesion to the fiber matrix but also establishes complex cross-trophic networks by enhancing physical connections and metabolic coupling between microorganisms, which are crucial for the stability of the microbial community (Gharechahi et al., 2020). Current research primarily focused on a few isolated strains from the Firmicutes and Fibrobacteres, such as *Ruminococcus flavefaciens* (*R. flavefaciens*), *Ruminococcus albus* (*R. albus*), and *Fibrobacter succinogenes* (*F. succinogenes*); however, these strains represent only a small fraction of the rumen microbial community (Hungate, 1950; Russell et al., 2009). Uncultured microbial groups, mainly comprising Firmicutes and Bacteroidetes, may provide new sources of CAZymes and offer insights beyond the traditional glycolytic pathways (Konietzny et al., 2014). Although most known mechanisms of lignocellulose degradation are based on classical models involving cellulosomes, secreted cellulases, and polysaccharide utilization loci (PULs), the cellulose degradation pathways in various microorganisms, particularly uncultured ones, remain incompletely understood. Exploring these unknown mechanisms is crucial for advancing our understanding of lignocellulose degradation and developing novel enzyme resources.

#### 4.1.1 Mechanism of lignocellulose degradation by Bacteroidetes

**Figure 3:**
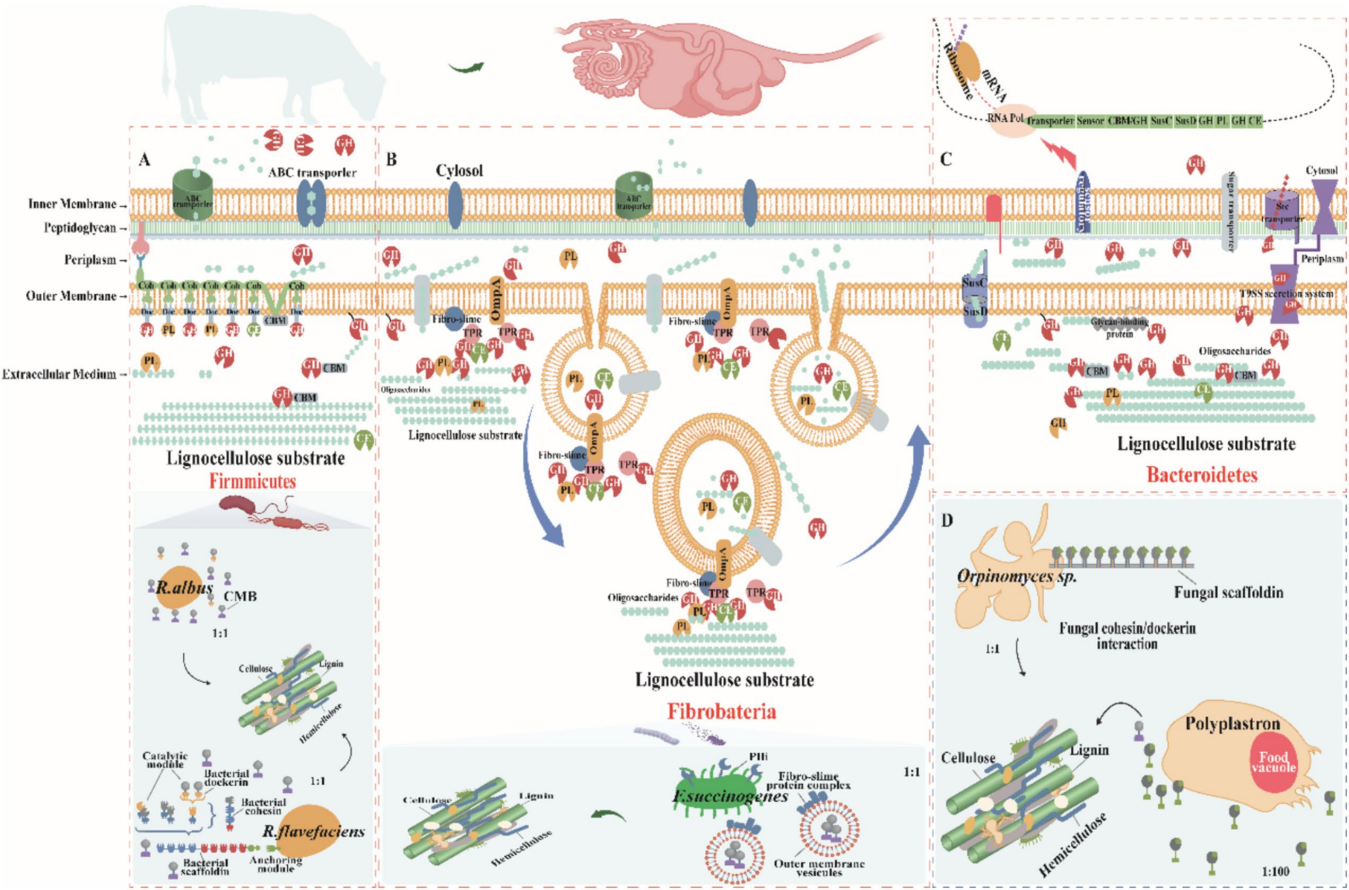
Mechanisms of lignocellulose degradation in ruminant gastrointestinal tract (GIT)microbes. **(A)** Firmicutes: Species such as *Clostridia* and *Ruminococcus* (including *R. flavefaciens* and *R. albus*) feature cellulosomes on their cell surfaces—complexes made of multidomain scaffolding proteins with cohesin and dockerin domains for CAZyme attachment, and carbohydrate-binding modules (CBMs) for targeting polysaccharides. Cellulosomes facilitate the hydrolysis of cellulose, with oligosaccharides then transported into cells via ATP-binding cassette (ABC) transporters for metabolism. Firmicutes also secrete extracellular enzymes to degrade less accessible substrates. **(B)** Fibrobacteria: *Fibrobacter succinogenes* binds cellulose using surface-localized proteins such as fibro-slime proteins and outer membrane protein A (OmpA), aggregating CAZymes to enhance degradation. This species employs the Type IX Secretion System (*T9SS*) for protein secretion and uses membrane vesicles loaded with CAZymes to access and degrade plant cellulose efficiently, enhancing metabolic integration. **(C)** Bacteroidetes: Polysaccharide degradation genes in Bacteroidetes are organized into PULs, which include SusC/SusD transport genes, CAZymes, and a transcriptional regulator, collectively regulated. Expression is induced by substrate presence, and periplasmic hydrolases further process oligosaccharides into monosaccharides for metabolism. Bacteroidetes also secrete CAZymes extracellularly via T9SS, facilitating direct substrate interaction. **(D)** Fungi and Protozoa: Organisms like *Orpinomyces* and *Polyplastron* employ unique mechanisms for lignocellulose degradation. *Orpinomyces* uses structures akin to bacterial fibrous vesicles, while Polyplastron produces GHs within specialized food vesicles, enhancing enzymatic activity. These diverse mechanisms highlight the complex and specialized strategies employed by ruminant GIT microbes to degrade lignocellulose, facilitating efficient nutrient extraction and host sustenance. Adapted from Moraïs and Mizrahi (2019) and Gharechahi et al. (2023).

Bacteroidetes dominate the GIT bacterial community, making up over 50% of rumen bacteria and excelling in polysaccharide degradation (McKee et al., 2021). They efficiently break down complex polysaccharides like lignocellulose and regulate enzyme expression to utilize carbon sources effectively (Lapébie et al., 2019). Specific gene clusters, such as the SusC/SusD, help them thrive in the rumen by encoding key proteins for PULs (Figure 3C; Bjursell et al., 2006). Although PULs were first found in Bacteroidetes, they are now common in other bacterial phyla like Proteobacteria, Planctomycetes, and Actinobacteria (Ausland et al., 2021), which contain enzymes like GHs, polysaccharide lyases, and carboxylesterases, enabling bacteria to break down specific polysaccharides (Grondin et al., 2017; Lapébie et al., 2019). *Bacteroides thetaiotaomicron*, for instance, uses multiple PULs to degrade complex polysaccharides such as rhamnogalacturonan II, with enzymes spread across three PULs (Ndeh et al., 2017; Wardman et al., 2022). The genetic structure and flexibility of PULs give Bacteroidetes an adaptive edge in nutrient-poor environments by optimizing enzyme expression and enhancing competitiveness in diverse microbial settings like the rumen. PUL expression is regulated by three main pathways: ECF-σ and anti-σ-factor pairs, SusR proteins, and a hybrid two-component system (Grondin et al., 2017). In the absence of substrate, the expression of PULs is inhibited and activated only in the presence of the target polysaccharide, thus ensuring efficient resource utilization (Pereira et al., 2021). During polysaccharide degradation, membrane-bound endoglucanases first hydrolyze the polysaccharide to produce oligosaccharides. Macrogenomic sequencing data indicate that Anabaena phylum occupies more than 60% of the total number of CAZymes in the rumen together with nitrogen-fixing bacteria (Gharechahi et al., 2021, 2022; Sharker et al., 2023). These bacteria have a wide range of degradation potential, efficiently degrading soluble sugars, glycoproteins, and a wide range of cell wall polysaccharides, and achieving efficient degradation through the synergistic action of PULs with free enzymes (Grondin et al., 2017; Lapébie et al., 2019). However, Bacteroidetes have a relatively limited role in cellulose degradation. While GH5 and GH9 family enzymes in some PULs have been implicated in cellulose degradation (Mackenzie et al., 2015), where cellulose is the only carbon source, these genes are not activated, suggesting that they may only play a role in hemicellulose degradation. Cellulose degradation is more dependent on free enzymes. For example, *Cyotophaga hutchinsonii* and *Sporocytophaga myxococcoides* degrade cellulose via free enzymes (McKee et al., 2021; Taillefer et al., 2018). Similarly, *Candidatus Paraporphyromonas polyenzymogenes* in the GIT of ruminants use a similar mechanism to degrade lignocellulose (Naas et al., 2018). The T9SS system, which is widespread in Gram-negative bacteria, has aided in the secretion of multidomain CAZymes, which enhances the degradation of complex cellulose and non-cellulosic substrates (Gao et al., 2022). In summary, the Bacteroidetes exhibits remarkable adaptive advantages in diverse carbon source environments through its unique enzyme secretion and regulation mechanisms, especially in lignocellulose degradation. The T9SS system is widely present in Gram-negative bacteria, helping them to secrete multidomain CAZymes that enhance the degradation of complex cellulose and non-cellulose substrates (Veith et al., 2017; Gao et al., 2022). Taken together, the phylum Anabaena exhibits significant adaptive advantages in diverse carbon source environments through its unique enzyme secretion and regulation mechanisms, especially in lignocellulose degradation.

#### 4.1.2 Mechanisms of lignocellulose degradation by the Firmicutes

Firmicutes degrade plant cell wall polysaccharides by two major enzyme systems: free enzymes and cellulosomes. Cellulosomes are multi-structural domain protein complexes consisting of multiple enzyme complexes capable of synergistically breaking down complex lignocellulose (Lamed et al., 1983). First described in the anaerobic *thermophilic bacterium Clostridium thermocellum*, cellulosomes are composed majorly of scaffolding proteins containing multiple cohesin structural domains that promote enzyme aggregation and functional synergism by interacting with the dockerin structural domains of cellulose-degrading enzymes (Artzi et al., 2017). This structure allows cellulosomes to efficiently degrade crystalline cellulose and other complex polysaccharides. In a typical cellulosome, the scaffolding protein subunit also contains additional dockerin and cellulose-binding modules (CBMs) for attachment to other cellulosomes and plant polysaccharide substrates (Artzi et al., 2017). The enzymes integrated into the cellulosomes vary from species to species and typically include critical cellulases and hemicellulases such as the GH5, GH9, GH10, GH11, GH26, and GH48 families. These enzymes create conditions for the degradation of cellulose microfibrils by removing polysaccharides such as xylan, mannan, xyloglucan, and pectin from the matrix (Gharechahi et al., 2021; Stewart et al., 2018, 2019). Within the Firmicutes, the *Ruminococcus* (e.g., *R. flavefaciens* and *R. albus*) are crucial cellulose-degrading bacteria capable of producing cellulosomes and secreting a wide range of secreted GHs to achieve the degradation of a wide range of polysaccharides. Most of these enzymes are multimodular in structure, incorporating multiple catalytic or non-catalytic structural domains (Arntzen et al., 2017). Within the Firmicutes, the genus *Ruminococcus* (e.g., *R. flavefaciens* and *R. albus*) is the key cellulose-degrading bacterium, capable of producing the cellulosome and secreting a wide range of secreted GHs to achieve degradation of a wide range of polysaccharides. Most of these enzymes are multimodular in structure, incorporating multiple catalytic or non-catalytic structural domains. However, the fact that *R. albus* does not produce a typical cellulosome despite the abnormal abundance of the CBM37 structural domain in its genome suggests that its cellulose degradation may depend on a different mechanism (La Reau and Suen, 2018; Artzi et al., 2017).The high expression of CBM37 in *R. albus* is associated with its cellulose-rich environments correlates with the adaptation of CBM37, which is thought to perform a vital role in substrate binding and cell surface localization of enzymes (Christopherson et al., 2014). It has been shown that *R. albus* 8 and *R. flavefaciens S85* preference type IV hyphae and carry CBM37 or cellulose glycosome CAZymes, respectively (Yeoman et al., 2021), whereas *R. albus* mutants lacking pivotal endoglucanases (Cel48A and Cel9B) are unable to adhere efficiently to cellulose surfaces, exhibiting defective cellulose degradation, further supporting the importance of CBM37 in cellulose degradation (Devillard et al., 2004). It has been shown that *R. albus* 8 and *R. flavefaciens* S85 prefer type IV hyphae and carry CBM37 or cellulosic glycosome carbohydrate-active enzymes, respectively (Yeoman et al., 2021), whereas *R. albus* mutants lacking the key endoglucanases (Cel48A and Cel9B) are unable to adhere effectively to cellulose surfaces, exhibiting defective cellulose degradation, further supporting the importance of CBM37 in cellulose degradation (Devillard et al., 2004). Based on these findings, a model of cellulose degradation by *R. albus* was proposed, emphasizing the synergistic roles of cell surface-localized CBM37 enzymes, cilium-like proteins, and extracellular glycocalyx matrices in cellulose matrix attachment and degradation (La Reau and Suen, 2018). However, the mechanisms involved in cellulose degradation in the Firmicutes are not fully understood. While the cellulosome performs an important role in some species, other species such as *C. albicans* lack typical scaffolding proteins and their cellulose degradation may rely on free enzymes or other unspecified mechanisms. This suggests that the Firmicutes may utilize diverse degradation strategies in response to different environments and substrates, an area where further research is still needed to fully understand the diversity and complexity of their cellulose degradation.

Firmicutes achieve efficient lignocellulose degradation by multiple mechanisms, relying not only on enzymes carrying dockerin structural domains integrated into the cellulosome, but also encoding a variety of independently acting CAZymes (Figure 3A; Gharechahi et al., 2023). These enzymes contain additional structural domains, such as CBMs, the latter of which perform a pivotal role in the degradation of lignocellulose by enhancing the enzyme’s substrate-specific binding (Artzi et al., 2017). In some cases, a single polypeptide has the capacity to carry multiple structural domains that dramatically enhance the degradation efficiency of the enzyme through synergistic structure–function interactions. For example, the fusion of the xylanase structural domain with the feruloyl esterase structural domain enables rapid cleavage of lignocellulosic hemicelluloses and their cross-linked lignin composite structures (Bayer et al., 2013). Firmicutes such as *Roseburia intestinalis*, a CBM35-containing CE17 enzyme catalyzes the deacetylation of β-mannan, making the mannan backbone more susceptible to degradation by GHs (Michalak et al., 2020). In this process, CBM35 improves the catalytic efficiency of the enzyme by targeting the acetylated substrate. Interestingly, CE17 often acts synergistically with CE2, co-encoded by specific PULs that catabolize mannans (La Rosa et al., 2019). This PUL is one of the typical structural features of gpPULs in Gram-positive bacteria and usually consists of a glucan transporter, multiple CAZymes and a transcriptional regulator. Moreover, in some enterobacteria, the mannan PUL encodes a GH26 mannanase localized on the cell surface that degrades mannans to oligosaccharides. These oligosaccharides then enter the cytoplasm via the ATP-binding cassette (ABC) transporter and are further deacetylated and depolymerized by the enzymes CE2 and CE17 for use in subsequent metabolic pathways (La Rosa et al., 2019).

*Ruminococcus albus* and *R. flavefaciens* are the major cellulose-degrading bacteria that use different strategies to efficiently degrade crystalline cellulose (Liang et al., 2024). For instance, *R. flavefaciens* assembles complex multi-enzyme complexes in the cell wall to degrade cellulose (Figure 3A; Liang et al., 2024), whereas *R. albus* enhances the efficiency of cellulose degradation through cellulosomes and enzymes containing CBMs 37 (Dassa et al., 2014). In terms of hemicellulose degradation, *Prevotella*, *Butyrivibrio*, and *Pseudobutyrivibrio* are the key genera of the Firmicutes. *Prevotella* is dominant in hemicellulose-rich environments, not only efficiently degrading hemicellulose, but also participating in the degradation of starch and proteins in the rumen (Stewart et al., 2018). A large-scale survey of the rumen microbiota of 1,000 dairy cows showed that 80–100% of the rumen microbiota contained these core genera (Wallace et al., 2019). These core genera (e.g., *Clostridium*, *Treponema*, *Eubacterium*, *Olsenella*, and *Oribacterium*) synergistically promote lignocellulose degradation and carbohydrate utilization by secreting large amounts of CAZymes (Moraïs and Mizrahi, 2019; Stewart et al., 2019). In addition, low abundance genera in the rumen microbiota also performed important roles in lignocellulose degradation and VFAs production. For instance, although *Succinivibrio* typically represents only 0.5% of the total bacterial population (Mizrahi and Jami, 2021), it functions as a cellulolytic bacterium capable of efficiently fermenting lignocellulose into organic acids via outer membrane vesicles, which serve as a substrate for other core microorganisms (Figure 3A). This function highlights the importance of low-abundance genera in the rumen microbial ecosystem. For instance, although Succinivibrio typically represents only 0.5% of the total bacterial population (Kim et al., 2011; Mizrahi and Jami, 2021), as a cellulolytic bacterium, it is able to efficiently ferment lignocellulose into organic acids via outer membrane vesicles, which provide substrates for other core microorganisms. This function highlights the importance of low abundance genera in the rumen microbial ecosystem.

#### 4.1.3 Mechanisms of lignocellulose degradation by the Fibrobacter

As part of the Fibrobacterota (Figure 3B), the *Fibrobacter* is the only well-defined group. The genus embraces two rumen-resident species with distinctive features, *Fibrobacter succinogenes* and *Fibrobacter intestinalis*, which have gained widespread attention in the ruminant digestive system due to their remarkable cellulolytic capacity (Kobayashi et al., 2008). These bacteria have evolved a unique cellulose degradation strategy that distinguishes them from other bacteria that rely on free enzymes or cellulosomes (Figure 3B). Notably, they possess neither PULs nor the structural basis of cellulosomes (Han et al., 2023). Of these bacteria, cellulases lack the typical cellulose-binding structural domains (e.g., CBM1, CBM2, and CBM3) that are common in other cellulose-degrading bacteria (Suen et al., 2011). However, their hemicellulases typically contain the CBM6 and CBM35 structural domains. These findings suggest that these bacteria employ an alternative and highly conserved cellulose degradation mechanism (Neumann and Suen, 2018). Bacteria of the *Fibrobacter* employ a cellulose degradation strategy that involves the aggregation of lignocellulose-degrading enzymes at the cell surface or the release of these enzymes into the environment via outer membrane vesicles, a strategy that has shown great efficiency in degrading complex cellulose structures (Gharechahi et al., 2023). Specifically, *Fibrobacter succinogenes* is not producing cellulosomes nor secreting free enzymes, but is directly degrading cellulose and other polysaccharides by producing outer membrane vesicles containing a variety of fiber-degrading proteins (Groleau and Forsberg, 1981; Gong and Forsberg, 1989). In the outer membrane of *F. succinogenes*, the only proteins capable of binding cellulose are fibrillar mucin and type IV bacteriorhodopsin (Figure 3B) (Burnet et al., 2015; Suen et al., 2011). When *F. succinogenes* strain S85 grows on a cellulose substrate, it produces membrane vesicles capable of degrading phosphate-swollen cellulose and carboxymethylcellulose (Groleau and Forsberg, 1981). These vesicles are detached from the bacterial outer membrane, have a spherical phospholipid bilayer structure, and contain lipopolysaccharides, membrane proteins, and proteins from the periplasmic space, as shown in Figure 3B (Arntzen et al., 2017). Recent studies have analyzed these vesicles by proteomics and found that they contain more than 300 proteins, of which about 21% have fiber-degrading functions (Arntzen et al., 2017). When the aforementioned membrane vesicles were added to *Panicum virgatum* biomass, they remarkably enhanced the catalytic efficiency of the commercial cellulase complex, boosting cellulase activity up to 2.4-fold of the basal level (Arntzen et al., 2017). Recent membrane proteomics analysis of *F. succinogenes* grown on cellulose as the sole carbon source revealed that cellulose-degrading enzymes are selectively localized to the cell membrane (Raut et al., 2019). Further analysis revealed that surface-exposed glycohydrolases contain a T9SS localization signal at their C-terminus, guiding their transport to the bacterial outer membrane. Following membrane translocation, the signal peptide is cleaved by proteases and the mature enzyme covalently binds to serine residues on the outer membrane (Raut et al., 2019). This membrane localization significantly enhances the binding of the bacteria to the substrate, thereby increasing the ability of the bacteria to engage with the cellulose substrate and its degradation products. The aggregation of outer membrane protein A (OmpA), fibrillar mucin, and tetrapeptide repeat -containing structural domains in the outer membrane of *F. succinogenes* when grown on a cellulose substrate suggests that they may be assembling cellulose-degrading enzymes at the cell surface, similar to the cellulosomes that form in the gram-positive bacterium *C. thermocellum* (Arntzen et al., 2017; Raut et al., 2019). TPR structural domain proteins are known to act as adapters to facilitate the interaction of other proteins, a role similar to that of scaffolding proteins in cellulosomes (Arntzen et al., 2017; Zeytuni and Zarivach, 2012). The cellulose-binding capacity of fibronectin may facilitate the attachment of bacteria or their membrane vesicles to polysaccharide substrates (Suen et al., 2011). The OmpA structural domain was recently found in a pectin cleaving enzyme, which is specifically enriched in the membrane vesicles of *F. succinogenes* when it grows on cellulose as a substrate (Arntzen et al., 2017). The OmpA structural domain contributes to the attachment of proteins carrying it to the peptidoglycan layer (Figure 3B). As shown in the model shown in Figure 3B, the bacteria direct the cellulose degradation machinery deeply into the substrate through membrane vesicles for efficient invasion of the substrate. Enzymes within the vesicles degrade the polysaccharide and the degradation products accumulate. Subsequently, the vesicles fuse with the cell membrane to bring the degradation products back into the cell. Previous studies have shown that *Fibrobacter succinogenes* is one of the most efficient bacteria for fiber degradation in the rumen (Dehority, 2003) and it is considered to be the most abundant of the major cellulose-degrading bacteria in most studies (Kobayashi et al., 2008). These findings not only emphasize the central role of Fibrobacter in rumen cellulose degradation, but also reveal the ecological and application potential of its unique degradation mechanism.

### 4.2 Degradation of lignocellulose by fungi in the GIT of ruminants

Gastrointestinal fungi of ruminants exhibit notable fiber degradation potential due to encoding a variety of plant fiber-degrading enzymes. Compared to prokaryotes (bacteria and archaea), these fungi are able to penetrate plant cell walls and dissolve lignin components more efficiently, and the phenolic compounds they produce are not metabolized by the organisms, leading to a weakening of the plant cell wall structure (Akin and Borneman, 1990). The abundance of anaerobic fungi is considerably lower than that of bacteria and protozoa, but their degradation capacity is higher than that of bacteria and protozoa (Hagen et al., 2021), and degrade approximately 20% of the coarse feed (Liang et al., 2021). Anaerobic fungi penetrate the plant cell wall through their rhizosphere system and cause physical damage to the lignocellulose structure, which in turn facilitates bacterial invasion and degradation (Swift et al., 2021), but lignin solubilization has been found to be extremely limited under anaerobic conditions (Susmel and Stefanon, 1993). Lignin forms a physical barrier that interferes with microbial degradation of cellulosic polysaccharides by covalently linking to the ferulate bridge of hemicellulose. Thus, fungi perform a critical role in degrading large particles in the early stages of degradation, increasing the exposure and degradation of plant cell wall polysaccharides by rumen bacteria (Dehority, 2003; Moraïs and Mizrahi, 2019; Lankiewicz et al., 2023). It has been found that anaerobic fungi degrade polysaccharides by producing a wide range of polysaccharide-degrading enzymes (Dehority, 2003; Moraïs and Mizrahi, 2019), especially *Neocallimastigomycota from Neocallimastix*, *Piromyces*, *Cacomyces*, *Anaeromyces*, *Orpinomyces*, and *Cyllamyces*, contributed significantly to this process (Fliegerova et al., 2015; Saye et al., 2021). The activity of these fungi is not limited to cellulose; they also efficiently decompose hemicellulose, with *Neocallimastix* and *Piromyces* showing the highest degradation efficiency (Fliegerova et al., 2015). Genomic analyses of anaerobic fungal species reveal a gene pool enriched in hemicellulases and cellulases (Youssef et al., 2013), with GHs presumed to have been acquired through bacterial-level gene transfer (Figure 3D; Haitjema et al., 2017). Fungal “cellulosomes” differ significantly from bacterial ones, with their dockerin module and putative scaffolding proteins, the adhesin module, being sequence- and functionally independent from bacterial cellulosomes (Haitjema et al., 2017), suggesting that their scaffolding systems are widely conserved in the anaerobic phylum of fungi. Also, they are allowed to undergo interspecies interactions (Fanutti et al., 1995). Moreover, *Neocallimastix frontalis* and *Meth anobrevibacter ruminantium* co-cultures exhibit high cellulase activity and CH4 yield (Saye et al., 2021). Addition of anaerobic fungal cultures to ruminant diets dramatically improved feed intake, growth rate, feed conversion efficiency and milk production. These fungi increase feed digestibility by improving gastrointestinal fermentation characteristics as well as enhancing microbial communities and cellulase activity (Kumar et al., 2015). However, difficulties in the culture and use of these strictly anaerobic fungi limit their potential for application in improving rumen fiber digestibility. However, difficulties in the culture and use of these strictly anaerobic fungi limit the possibility of practical applications to enhance fiber digestion in the rumen through feeding.

### 4.3 Degradation of lignocellulose by protozoa in the GIT of ruminants

Protozoa perform a central role in the ruminant gastrointestinal ecosystem and consist mainly of ciliates such as *Dasytricha ruminantium*, *Entodinium*, and *Epidinium* (Finlay et al., 1994; Henderson et al., 2015). These protozoa not only promote the decomposition of plant tissues, but also synergistically break down lignocellulose by secreting enzymes such as GHs (Figure 3D; McKee et al., 2021). They function in the early stages of fiber colonization and promote further fiber degradation by consuming low concentrations of oxygen to reduce the impact of oxygen in the rumen (Gruninger et al., 2019). Metatranscriptomic studies have revealed a wide range of CAZymes in protozoa (Williams et al., 2020), while activity-based metagenomic screens have identified numerous cellulolytic enzymes (Xie et al., 2021; Cao et al., 2023; Wu et al., 2024). In particularly, the ability of some protozoans such as *Eudiplodinium maggii* to directly ingest lignocellulose highlights their critical in fiber degradation. In spite of their dependence on exogenous sources of nitrogen in nitrogen metabolism, protozoa acquire amino acids by phagocytosing bacteria and fungi to stimulate the growth of novel rumen bacteria, which promotes the degradation of low concentrations of dietary fiber (Huws et al., 2018). In addition, the symbiotic relationship between protozoa and methanogens significantly affects methane production, with hydrogen produced by protozoa being utilized by methanogens to convert CO₂ to CH₄, which in turn promotes protozoan proliferation (Solomon et al., 2022). This interaction suggested that the microbial dynamics of rumen anaerobic digestion can be indirectly regulated by modulating the protozoan community to optimize productivity (Newbold et al., 2015). While current research was focused on protozoa in the rumen, less research has been conducted on their presence and role in the gut. However, these protozoa contribute vitally to the breakdown of a wide range of carbon sources (e.g., starch, cellulose, hemicellulose, and pectin), accounting for approximately 50% of the rumen biomass and significantly influencing the metabolism of the ecosystem (Wang and McAllister, 2002; Newbold et al., 2015). Henderson et al. (2015) found that rumen protozoa were categorized into 12 distinct species in 742 samples covering 35 countries and 32 animal species, with the genera *Entodinium* and *Epidinium* appearing in 90% of the samples and accounting for 54% of the sequencing data. Even though some protozoa exhibit remarkable cellulolytic activity, the genomes of the most cellulolytic species like *Eudiplodinium maggii* and *Epidinium ecaudatum* have not yet been sequenced, limiting a deeper understanding of their function (Dehority, 2003). Removal of protozoa reduced the digestibility of neutral and acidic detergent fibers by 20 and 16%, respectively, although some fiber digestibility was restored in the hindgut (Wang and McAllister, 2002; Newbold et al., 2015). The degradation of lignocellulose is a complex process that requires the synergistic action of multiple fiber-degrading microorganisms, with protozoa playing a key role in the fiber colonization and initial digestion stages. This suggests that the removal of protozoa not only affected fiber degradation, but also exerted remarkable effects on the overall microbial community, for instance a dramatic decrease in fungal concentrations and a sharp increase in the number of fiber-degrading bacteria. Furthermore, controversy still exists regarding the effect of protozoan removal on feed conversion. In conclusion, the study of gastrointestinal protozoa faces multiple challenges, which limit a comprehensive understanding of their mechanisms of action, but an in-depth study of these microorganisms is important for unraveling their functions, developing rumen-specific protozoa to improve host phenotypes, and minimizing environmental impacts. Therefore, systematic studies on gastrointestinal protozoa should be intensified in the future to facilitate the development of ruminant biology and related applications (Table 2).

**Table 2:** Fungal and protozoan species on lignocellulose.

Microbiology	Lignocellulose fraction	Products	Literatures
Fungi			
*Anaeromyces*	Cellulose	Cellobiose	Rabee et al. (2019)
*Neocallimastix coliforniae*	Cellulose	Cellobiose	Rabee et al. (2019)
*Neocallimastix*	Cellulose/hemicellulose	Cellobiose/pentose	Solomon et al. (2016)
*Piromyces finnis*	Cellulose	Cellobiose	Rabee et al. (2019) and Huan (2024)
*Cyllamyces*	Cellulose	Cellobiose	Solomon et al. (2016)
*Orpinomyces* sp.	Cellulose	Cellobiose	Borneman et al. (1989)
*Pecoramyces*	Cellulose	Cellobiose	Li et al. (2022)
*Neocallimastir patricianun*	Hemicellulose	Pentose	Froman et al. (1989)
*Hypocre* sp.	Hemicellulose	Pentose	Li et al. (2007)
*Kluyveromyces* sp.	Hemicellulose	Pentose	Juturu and Wu (2012)
*Ascomycota*	Lignin	Hexose sugar	Liang et al. (2020)
*Basidiomycota*	Lignin	Hexose sugar	Han et al. (2023)
Protozoa			
*Epidinium*	Hemicellulose/cellulose	Pentose/cellobiose	Han et al. (2023)
*Polyplastron*	Hemicellulose	Pentose	Han et al. (2023)
*Diplodiniinae*	Hemicellulose/cellulose	Pentose/cellobiose	Li et al. (2022)
*Ophryoscolecinae*	Hemicellulose/cellulose	Pentose/cellobiose	Solomon et al. (2022)

## 5 Degradation patterns of lignocellulose by GIT microbial enzymes in ruminants

**Figure 4:**
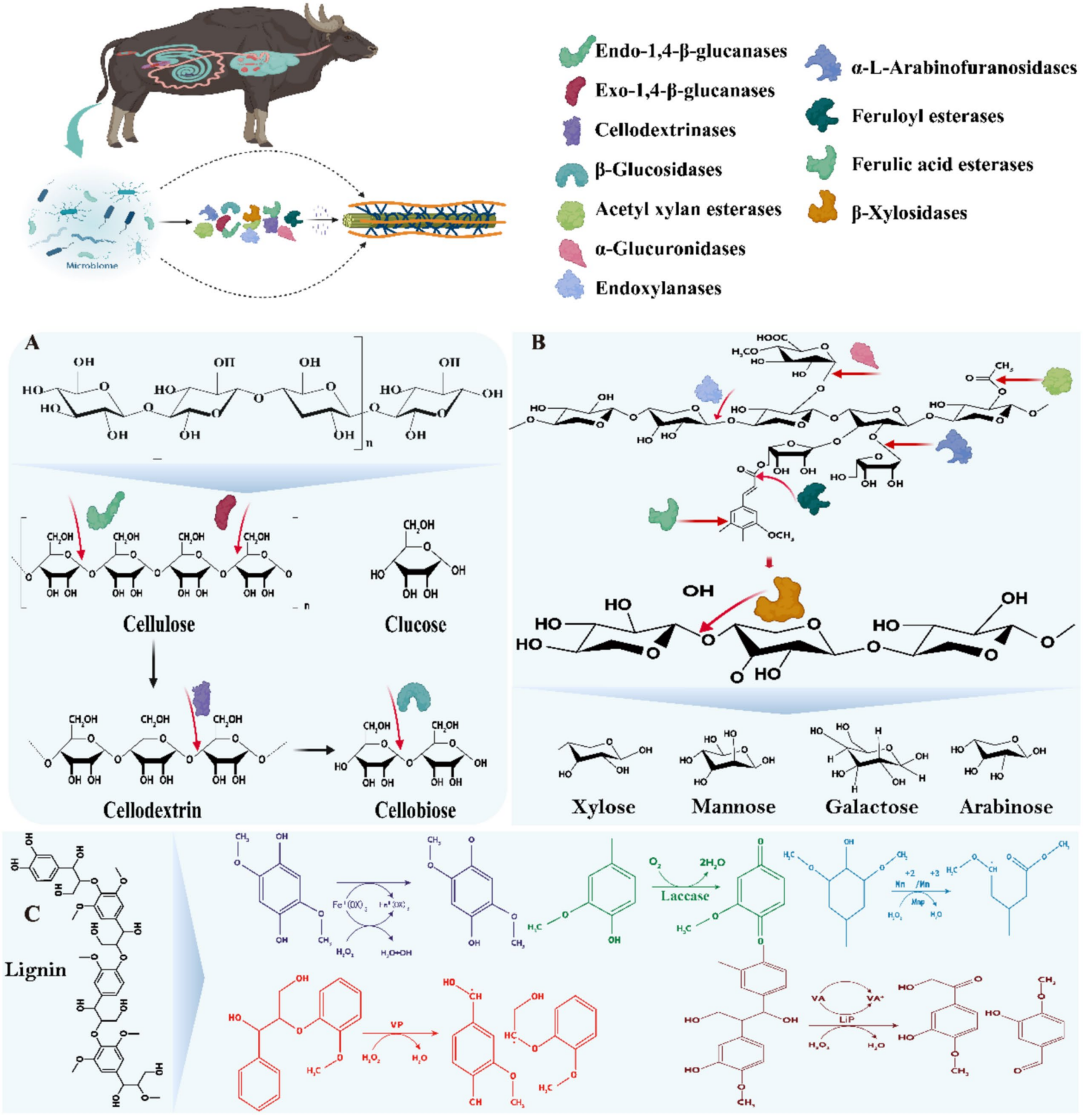
Mechanisms of action of microbial enzymes in the GIT of ruminants mainly involved in the degradation of lignocellulose. **(A)** Mechanisms of action of cellulase mixtures; **(B)** Mechanisms of action of hemicellulases/xylanases; **(C)** Mechanisms of action of ligninases/laccase. The substance was adapted and modified from Rajeswari et al. (2021).

Cellulose microfibrils are embedded in a matrix of hemicellulose (mainly xylan, but also mannan, xylan, and β-glucan), lignin, and pectin in the plant cell wall. The cellulose component is crystalline and insoluble in water. Therefore, in order to achieve efficient degradation of plant fibers in the GIT of ruminants, multiple cellulases, hemicellulases, and ligninases are required to act synergistically on different portions of the composite biopolymer (Figure 4). Fiber degradation functions are expressed by a variety of known enzymes (e.g., cellulases and hemicellulases), which are clustered into approximately 150 GH families classified according to their sequence, functional, and structural properties (Lombard et al., 2014). In the following, we described the microbial enzyme functions that degrade lignocellulose in the GIT of ruminants.

### 5.1 Composition and degradation mechanism of cellulases

Cellulases are a group of complex enzyme systems capable of hydrolyzing the β-1,4-glycosidic bonds in cellulose, including major endoglucanases, exoglucanases and β-glucosidases (Liang et al., 2020). The structure of cellulose, the most abundant polysaccharide in plant cell walls, consists of β-1,4-glycosidically linked glucose, with inter- and intramolecular hydrogen bonding conferring a high degree of crystallinity and insolubility (Bhujbal et al., 2021; Liang et al., 2020). Among these enzyme systems, endoglucanases are mainly encoded by the GH5-9, GH12, GH44-45, GH48, GH51, GH74, and GH124 families, exoglucanases are classified as type I and type II and are encoded by GH5-6, GH9, and GH48, GH51, GH74, and GH124, respectively, and β-glucosidases are encoded by GH1, GH3, GH5, GH9, GH30, and GH116 families (Bhujbal et al., 2022). In the GIT of ruminants, cellulases secreted by a variety of microorganisms act synergistically to hydrolyze cellulose through synergistic interactions, including endonuclease β-1,4 glucanase, which cleaves the internal bonds in the amorphous region; exonuclease β-1,4 glucanase, which cleaves either the reducing or non-reducing ends of cellulose molecules produced by endonuclease β-1,4 glucanase; and β-glucosidase, which hydrolyses the exonuclease β-1, 4 glucanase product into a single monosaccharide by hydrolysis (Figure 4A; Liang et al., 2020; Lin et al., 2023). These microbial populations are enriched with CAZymes genes that play key roles in carbohydrate degradation, involving classes such as GHs, CEs, GTs, CBMs, PLs, and AAs (Xie et al., 2021; Glendinning et al., 2021; Lin et al., 2023; Jin et al., 2024), of these, Bacteroidetes, Firmicutes, Fibrobacteres and Planctomycetes contributed the most. In cellulose degradation, the GH48-expressing family such as the genus *Ruminococcus* is particularly prominent, whose genome contains at least one GH48 gene with the ability to degrade crystalline cellulose (Moraïs and Mizrahi, 2019). In contrast, cellulolytic fungi exhibit a more diverse range of GH families, with the GH6 family in particular showing efficient capacity (Liang et al., 2020; Hagen et al., 2021). However, the accumulation of fiber biosaccharides often inhibits the activities of endoglucanase and exoglucanase, reducing the degradation efficiency. To overcome this challenge, Hooker et al. (2018) found that *Piromyces* sp. *UH3-1* significantly increased β-glucosidase production, which enhanced the cleavage rate of cellobiose. The classical model of enzymatic hydrolysis consists of (Figure 4) (1) endoglucanase randomly cuts the β-1,4-glycosidic bond within the amorphous region of the cellulose molecule, reducing the degree of polymerization of the cellulose molecule; (2) cellobiose hydrolases of types I and II act on the reducing and non-reducing ends of the cellulose molecule, respectively, to produce either glucose or cellobiose; and (3) β-glucosidases further hydrolysed to glucose (Rajeswari et al., 2021). In addition, soluble polysaccharide monooxygenase (LMPO) accelerates cellulose degradation by oxidatively breaking glycosidic bonds and acting synergistically with conventional cellulases (Houfani et al., 2020). Although existing studies have revealed the diversity of CAZyme in the gastrointestinal microbiota and its key role in cellulose degradation, several challenges remain. Firstly, the synergistic mechanisms among different species are not fully understood, especially in complex microbial communities, and how the synergistic effects of various enzymes can be efficiently performed in real feeding environments urgently needs to be thoroughly investigated. Secondly, the inhibitory effect of fiber biosaccharides accumulation on enzyme activity needs to be overcome by molecular engineering or screening of efficient enzyme lines. In addition, the functional The classical model of enzymatic hydrolysis consists of (Figure 4) (1) endoglucanase randomly cuts the β-1,4-glycosidic bond within the amorphous region of the cellulose molecule, reducing the degree of polymerization of the cellulose molecule; (2) cellobiose hydrolases of types I and II act on the reducing and non-reducing ends of the cellulose molecule, respectively, to produce either glucose or cellobiose; and (3) β-glucosidases further hydrolysed to glucose (Ferreira et al., 2021). In addition, soluble polysaccharide monooxygenase (LMPO) accelerates cellulose degradation by oxidatively breaking glycosidic bonds and acting synergistically with conventional cellulases (Houfani et al., 2020). Although existing studies have revealed the diversity of CAZyme in the gastrointestinal microbiota and its key role in cellulose degradation, several challenges remain. Firstly, the synergistic mechanisms among different species are not fully understood, especially in complex microbial communities, and how the synergistic effects of various enzymes can be efficiently performed in real feeding environments urgently needs to be thoroughly investigated. Secondly, the inhibitory effect of cellobiose accumulation needs to be addressed by molecular engineering or screening of efficient enzyme lines. In addition, the functional differences of CAZyme in the gastrointestinal tract of different ruminants suggest potential strategies to optimize the efficiency of carbohydrate utilization, but specific application options need to be further explored.

### 5.2 Composition and degradation mechanism of hemicellulases

Hemicellulose is the second major component of plant cell walls and has a complex structure consisting of various polysaccharides such as xylose, arabinose, and mannan, with xylose being the most abundant (Figure 4B; Bhujbal et al., 2021; Liang et al., 2020). Unlike cellulose, hemicellulose consists of xylose chains and their different side-chain sugars, which are linked by β-1,4-glycosidic bonds, conferring a high difficulty in hydrolysis. The breakdown of hemicellulose is mediated by a variety of CAZymes, such as xylanases, xylosidases, and mannanases, which cover the families GH5, GH10, GH11, GH26, GH30, GH43, and GH74 (Shen et al., 2020; Plouhinec et al., 2023). Of gastrointestinal microorganisms in ruminants, *Ruminococcus* and *Fibrobacter* are also capable of producing hemicellulases, although they are mainly involved in cellulose degradation. Macrotranscriptome analysis showed that two-thirds of the hemicellulases produced by *Ruminococcus*, *Prevotella*, and *Fibrobacter* belong to the GH10, GH11, and GH26 families (Dai et al., 2015). *Prevotella* and *Butyrivibrio* are regarded as the most potent hemicellulose-degrading genera due to their diverse enzymatic activities and rich enzyme libraries (Liang et al., 2020). Anaerobic fungi, such as *Anaeromyces*, *Neocallimastix*, *Orpinomyces*, and *Piromyces*, also play a key role in hemicellulose decomposition, with *Neocallimastix* and *Piromyces* in particular showing efficient degradation capabilities (Puniya et al., 2015; Gruninger et al., 2018). Genome sequence analysis revealed that hemicellulases are extremely abundant in these anaerobic fungi, despite the low abundance and limited information on the relevant reference sequences in the database (Youssef et al., 2013). It was also found that protozoa abundantly express CAZymes belonging to the GH5, GH9, GH10, GH11, and GH48 families, further confirming their key role in hemicellulose degradation (Liang et al., 2020). The hemicellulose structure also includes mannans and β-glucans, the hydrolysis of which requires additional GH family enzymes. For example, mannanases are mainly from the GH5 and GH26 families, xylanases are attributed to the GH5, GH44, GH74, and GH12 families, and β-glucanases are from the GH5, GH16, and GH17 families (Moraïs and Mizrahi, 2019). In the gastrointestinal tract of dairy cows, the GH10 and GH11 family genes are highly expressed and most hemicellulase sequences are encoded by Ruminococcus, Prevotella and Fibrobacter (Dai et al., 2015; Lin et al., 2023). *Neocallimastix frontalis* and *Piromyces rhizinflata* in these fungi showed significant expression of the GH10 and GH11 families at the transcriptional level, reflecting their potential efficient degradation capacity (Gruninger et al., 2018). Meanwhile, hydrolysis of mannan and β-glucan involves GH5, GH16, GH17, GH26, GH44, GH74, and GH12 families (Moraïs and Mizrahi, 2019). In addition, co-culture studies have shown that the interaction of anaerobic fungi with methanogens such as *Methanobrevibacter* dramatically augmented xylan degradation efficiency, suggesting that the efficiency of hemicellulose utilization can be improved by optimizing the microbial community (Leggieri et al., 2021). Although the diversity of hemicellulases and their key role in the gastrointestinal microbiota of ruminants have been recognized, the challenge of overcoming the complex synergistic mechanisms and inhibition of enzyme activity among these microorganisms, and the application of these mechanisms in practical feeding environments, remain important directions for future research.

### 5.3 Composition and degradation mechanism of ligninolytic enzyme

Lignin is the third major component of lignocellulose, which has a complex three-dimensional structure and consists of three different phenylpropane monomers - coniferyl alcohol, kaempferol and p-coumaryl alcohol. Studies have shown that gastrointestinal microorganisms can noticeably alter the fiber content by decomposing lignin, in which the lignin content can be reduced by 5–20% (Yue et al., 2013). In an *in vitro* fermentation study of wheat straw, rumen aerobic fungi, Ascomycota, Basidiomycota, and bacteria such as *Pseudomonas* and *Olsenella* were involved in lignin degradation by secreting ligninase (Xing et al., 2020). In addition, anaerobic fungi secrete esterases (e.g., p-coumaroyl esterase, acetyl esterase, and feruloyl esterase) to efficiently separate lignin from hemicellulose, thereby releasing more sugars for metabolism by other microorganisms (Figure 4C; Borneman et al., 1990). Nevertheless, the mechanism of lignin breakdown under anaerobic conditions is not fully understood. Therefore, there is an urgent need for current studies to explore in depth the lignin degradation process and its microbial mechanisms of action, especially the roles of key molecular and biochemical pathways, in order to better understand and utilize this process. This will not only help to optimize the conversion efficiency of woody biomass, but also has important implications for environmental protection and sustainable energy development.

## 6 Conclusion

This review comprehensively explored the central role of ruminant gastrointestinal microorganisms in plant fiber digestion and utilization. Although a limited number of microbial species have been isolated and cultured, these microbes collectively contribute to the complete hydrolysis of cell wall polysaccharides through the production of multiple synergistic hydrolytic enzymes. Advances in metagenomic analysis techniques have enabled us to gain a detailed understanding of the enzyme profiles encoded by these microorganisms during fiber degradation. The action of these enzymes is not limited to the intracellular compartment; many are secreted into the GIT or bound to the cell membrane/envelope to enhance their efficiency of action. Enzymes secreted by microorganisms are at risk of inactivation and protein degradation in the gastrointestinal environment, and their degradation products may be absorbed by other microorganisms or hosts. Therefore, lignocellulose-degrading bacteria evolved multiple mechanisms to minimize these risks, such as by localizing CAZymes to the cell surface, producing membrane-anchored enzyme complexes, and developing membrane transporters. Understanding of these mechanisms could help in the development of new probiotics or in improving feed digestion efficiency in animal production systems through microbiome engineering.

Despite our understanding of the microbial community in the gastrointestinal tract of ruminants, there still exists a large number of uncultured microorganisms whose functions and mechanisms of action are still unclear, which to some extent limits our comprehensive knowledge of the microbial ecosystem. In the field of enzymology research, although a variety of enzymes involved in lignocellulose degradation have been identified, the synergistic mechanism of enzyme action among different species is still not fully understood, while the inhibition of enzyme activity by degradation products such as fibrous disaccharides has not yet been effectively addressed. At present, the integration and application of multi-omics technology is not deep enough to fully reveal the microbial activities in the gastrointestinal tract. Based on these limitations, future research should focus on the following aspects. Firstly, strengthening the study of uncultured microorganisms, developing new culture techniques and methods, and exploring their role in lignocellulose degradation and their interrelationships with other microorganisms in depth. Secondly, combining multidisciplinary means such as molecular biology and biochemistry, in-depth research on the synergistic mechanism of enzymes, optimizing enzyme performance through genetic engineering and other techniques, and overcoming the problem of inhibition of enzyme activity by degradation products. Finally, we will further strengthen the integration and application of multi-omics technologies, comprehensively analyse the genomic, transcriptomic, proteomic and metabolomic data of microorganisms, and comprehensively analyse the metabolic pathways and regulatory mechanisms of microorganisms in the gastrointestinal tract. In addition, more *in vivo* and in vitro experiments can be carried out in the future to simulate different feeding conditions and environmental factors to study the dynamic changes of microbial communities and their effects on lignocellulosic degradation efficiency, so as to provide a more solid theoretical basis and practical guidance for the improvement of roughage utilization efficiency in ruminants.

## Footnotes

^1^ https://www.cazy.org
